# Gynaecology, general surgery and urology residents’ perspective and exposure to endoscopy training and practice: a cross-sectional study of resident doctors in four residency training centres in Abuja, Nigeria from June to August, 2020

**DOI:** 10.11604/pamj.2023.44.94.27521

**Published:** 2023-02-16

**Authors:** Azuka Chinweokwu Ezeike, Toochi Oliver Nwoye, Oladele Olasunkanmi Situ, David Tolulope Ejenobo, Febian Ehimatie Obakeye, Augustine Monday Onuh, Joseph Ifeanyichukwu Ikechebelu

**Affiliations:** 1 Obstetrics and Gynaecology Department, National Hospital Abuja, Abuja, Nigeria,; 2 Obstetrics and Gynaecology Department, Federal Medical Centre Abuja, Abuja, Nigeria,; 3 General Surgery Division, Surgery Department, National Hospital, Abuja, Nigeria,; 4 Obstetrics and Gynaecology Department, Nisa Premier Hospital Abuja, Abuja, Nigeria,; 5 Obstetrics and Gynaecology Department, University of Abuja Teaching Hospital Abuja, Abuja, Nigeria,; 6 Obstetrics and Gynaecology Department, Garki Hospital Abuja, Abuja, Nigeria,; 7 Obstetrics and Gynaecology Department, Nnamdi Azikiwe University Teaching Hospital Nnewi, Nnewi, Nigeria

**Keywords:** Endoscopy, training, exposure, residency

## Abstract

**Introduction:**

the use of endoscopy in the surgical management of a wide range of ailments has revolutionised the practice of surgery. Endoscopy however has been underutilised in developing nations. Optimal training exposure during the residency training program is considered as very crucial to the improvement of endoscopy practice in this region. The objective of this study was to evaluate the perception and endoscopy training exposure of resident doctors in gynaecology, general surgery and urology in four residency training centres in Abuja.

**Methods:**

this was an analytical cross-sectional study of endoscopy exposure of gynaecology, general surgery and urology resident doctors in four residency training centres in Abuja from June through August 2020. Using a structured questionnaire, information was obtained on demography, perception of endoscopy, exposure to and expectations for endoscopy training and practice. Data were analysed with SPSS version 25 (IBM Corp., Armonk, NY, USA).

**Results:**

a total of 125 questionnaires were distributed with a 92% response rate. The mean age of the respondents was 36.17±4.62 years with a mean duration of training of 53.91±28.02 months. Eighteen (15.8%) were satisfied with endoscopy practice in their centre and only five respondents (4.4%) had attained competence in operative endoscopy. Twelve trainees (10.5%) reported that they had received formal training in endoscopy outside their workplace and 109 (95.6%) desired to have post-fellowship training. Competence was statistically significantly higher among the senior registrars compared to registrars (Fisher 51.81, P=< 0.001). Lack of funding was the most reported limiting factor to endoscopy training (66.7%) while most (85.1%) desired the incorporation of structured endoscopy training into the residency training curriculum.

**Conclusion:**

this study showed poor endoscopy training exposure, a high level of dissatisfaction with the state of endoscopy practice and high expectations of the trainees for improved training facilities and human capacity.

## Introduction

Endoscopy is the use of minimally invasive techniques for diagnosis and treatment. Modern endoscopy came into the limelight in the late 20^th^ Century, since then, the practice has expanded and found relevance in almost all fields of Gynaecology and Surgery [1]. Laparoscopy and hysteroscopy are useful adjuncts in the management of infertility which has a high burden in this part of the world [2]. They are also relevant in the management of other gynecological ailments. In surgical practice, laparoscopy, upper and lower gastrointestinal tract endoscopy and other endoscopic procedures are viable alternatives in the management of general surgery and urology pathologies [3]. Endoscopy has the benefit of early recovery, early return to work and cosmetic advantages [3,4]. Considering the benefits, there is a justified increase in demand and to meet this demand, full-fledged endoscopy practice is essential. Competence in endoscopy can only be developed through structured training, so training is fundamental [5]. Training involves didactic lectures, video tapes, computer-generated programs and hands on activities including experience with pelvic trainers/animal models, assistance in the theatre and supervised surgeries [6]. To facilitate training there is a need for structured training programs enhanced by the presence of specialist endoscopy surgeons, facilities for endoscopy and endoscopy skills laboratory. This is a challenge in sub-Saharan Africa as there are limited facilities for formal training in endoscopy [2,4]. In the early 1970's, the John Hopkins's Programme for International Education in Gynaecology and Obstetrics (JHPIEGO) pioneered the introduction of laparoscopy training in sub-Saharan Africa, this was however not sustained over the years [7].

Residency training is a period of postgraduate training that spans about six years and provides a good opportunity for developing proficiency in endoscopy. The incorporation of endoscopy training into the residency program has been advocated as a way of improving competence [6,8]. Though the evidence of endoscopy practice is a requirement for accreditation of residency training centres by the Postgraduate medical colleges, yet endoscopy training has not been included in mainstream postgraduate (residency) training in most African countries and there are no structured programme guidelines. Inadequate facilities, absence of specialist endoscopy surgeons, lack of dedicated endoscopy units and theatre time have also been identified as obstacles to training [4,9,10]. Though there are a few training centres in Nigeria that offer short intensive endoscopy courses, participation is usually on a voluntary basis. Residents´ participation has not been optimal from factors ranging from lack of sponsorship to inability to secure leave from work to attend such courses. More advanced trainings are generally available outside the country at exorbitant costs. Even when residents are trained, facilities may not be available in their training centres for sustained practice [2,4]. This has hampered the advancement of endoscopy in Nigeria [2]. In a survey of postgraduate trainees in Nigeria, Balogun *et al*. found that though 80% had a desire for future endoscopy practice, up to 90.7% had received no formal training in laparoscopy [11].

Implementing endoscopy training has been a challenge in sub-Saharan Africa [2,10]. The poor state of modern surgical training in sub-Saharan Africa has been attributed to unstable government and institutional policies, dwindling national economy, low budgetary allocation, brain drain syndrome as well as poor facilities [12]. In developed countries however there are better structured but not yet optimal programmes [13,14]. Studies done in the United Kingdom (UK), Germany, Netherlands and Canada show better exposure of the residents to endoscopy training though some of the centres still lacked standardized postgraduate endoscopy training curricula and optimal training environment [13-16]. The lack of objective assessment has also been noted as a major hindrance to improvement in competence, even where training is available [17]. There is presently no published study on the exposure and expectations of gynaecology and surgical residents in Abuja, Nigeria to endoscopic surgery so this study set out to answer the research question; What is the perception and level of endoscopy training exposure of resident doctors in gynaecology, general surgery and urology in four centres in Abuja? This study aimed to appraise the perception and level of endoscopy training exposure of residents in gynaecology, general surgery and urology in four centres in Abuja. This survey serves as a needs assessment of the current status of endoscopy surgery training in Nigeria and this forms the rationale for this study. The findings therefore will form the basis for providing evidence-based recommendations to policy makers on the future of endoscopy training during the residency program.

## Methods

**Study design/sample size:** this was an analytical cross-sectional study and self-administered structured questionnaire was used to obtain information from consenting eligible gynaecology, general surgery and urology residents in the four residency training centres. The purposive sampling method was utilised for this study. One hundred and seventy-three (173) resident doctors having their residency training in the specified specialties during the study period were assessed for eligibility. Of this number, only 125 who met the inclusion criteria were recruited. This number determined the sample size.

**Study setting:** this study was done in four residency training centres in Abuja. Abuja is the administrative capital of Nigeria and is located within the North Central geo-political zone of the country. Participants were drawn from National Hospital Abuja, Federal Medical Centre Abuja, University of Abuja Teaching Hospital, Abuja and Garki Hospital, Abuja.

**Centre 1-**National Hospital, Abuja is a 450-bed capacity, federal government-owned tertiary hospital with an accredited residency training program in obstetrics and gynaecology and surgery since 2002.

**Centre 2-**Federal Medical Centre, Abuja is a 160-bed capacity federal government-owned tertiary hospital in Abuja with an accredited residency training program in obstetrics and gynaecology obtained in 2013 and surgery in 2019.

**Centre 3-**University of Abuja Teaching Hospital Gwagwalada is a 350-bed capacity, government-owned tertiary hospital with accredited residency training program in obstetrics and gynaecology obtained in 2002 and surgery in 2004.

**Centre 4-**Garki Hospital, Abuja is a 120-bed capacity secondary health facility operated under a public-private partnership and obtained joint residency accreditation with Nisa Premier Hospital Abuja (a 60-bed capacity private hospital) in obstetrics and gynaecology in 2011. It is yet to obtain residency training accreditation in surgery.

**Participants/eligibility criteria:** included in the study were registrars and senior registrars in gynaecology, general surgery and urology in the respective centres. Doctors with less than one year of training were excluded from the study.

### Data collection

Designated members of the research team distributed the questionnaires in each centre. Data were collected from June to August 2020. Majority 107 (85.6%) of the questionnaires were hand- delivered to the participants. A few, 18 (14.4 %) were mailed as electronic copies to trainees who were unavailable in the hospital as the study was done at the peak of the COVID-19 pandemic in Nigeria with the resultant disruption of clinical services. Informed consent was obtained from all respondents and confidentiality and anonymity were maintained. Relevant information was obtained using a structured self-administered questionnaire. The questionnaire has an introduction that highlighted the importance of the study to endoscopy practice, that the anonymity of the respondents will be maintained and the need for their consent. It consisted of four sections which included data on socio-economic variables, exposure to endoscopy practice, exposure to formal endoscopy training and expectations with respect to endoscopy training and practice. Socio-demographic data obtained included age, sex, duration of residency training, cadre and training centre. Other data obtained include perception of the relevance of endoscopy, satisfaction with the level of endoscopy practice in their centre, limiting factors to endoscopy practice, exposure to formal endoscopy training in the past, intentions for further endoscopy training, expectations from a training program, readiness to self-sponsor their endoscopy training, the average number of endoscopy surgeries in their centre per month and the presence of endoscopy skills lab. The perception of the relevance of endoscopy practice and degree of satisfaction of the residents were assessed on a five-point Likert scale.

### Data measurement/statistical analysis

The data were inputted into the Statistical Package for Social Sciences (SPSS) version 25 (IBM Corp., Armonk, NY, USA) Continuous variables were summarised as means and standard deviation while categorical variables were summarized as frequencies and proportions. A p-value of less than 0.05 was considered statistically significant. The objectives of the analysis were to: (a) Describe the general characteristics of the study population, (b) Compare the demographic characteristics of the respondents by specialty, (c) Evaluate the perception of the resident doctors of limiting factors to endoscopy practice and their expectations and (d) Compare the level of competence, perception of endoscopy training and level of satisfaction with endoscopy training of the resident doctors by specialty, training centre and cadre. Categorical variables were compared with Pearson´s Chi-square test, however where at least 25% of the cells have an expected count of less than five (5) Fisher´s exact test was utilised. A p-value of less than 0.05 was considered statistically significant.

To evaluate the exposure of the resident doctors to endoscopy training, we conducted the data analysis using these variables; the average number of endoscopy surgeries in their centre, the presence of skills laboratory, prior exposure to formal endoscopy training and level of endoscopy surgery competence. The results were interpreted as frequencies and proportions. Comparison of level of competence between gender and cadre was done with Chi-square test or Fisher´s exact test. To evaluate the perspective of the resident doctors of endoscopy training, we conducted the analysis using these variables; perception of the relevance of endoscopy training, level of satisfaction with endoscopy training and desire for post-fellowship training. The results were interpreted as frequencies and proportions and compared between specialties with Chi-square test or Fisher´s exact test. To evaluate the perception of the resident doctors of limiting factors to endoscopy practice and their expectations, we conducted the analysis using these variables; perceived limiting factors, expectations for endoscopy practice in their centre and expectations from a training program. The results were interpreted as frequencies and proportions.

## Results

**Figure 1:**
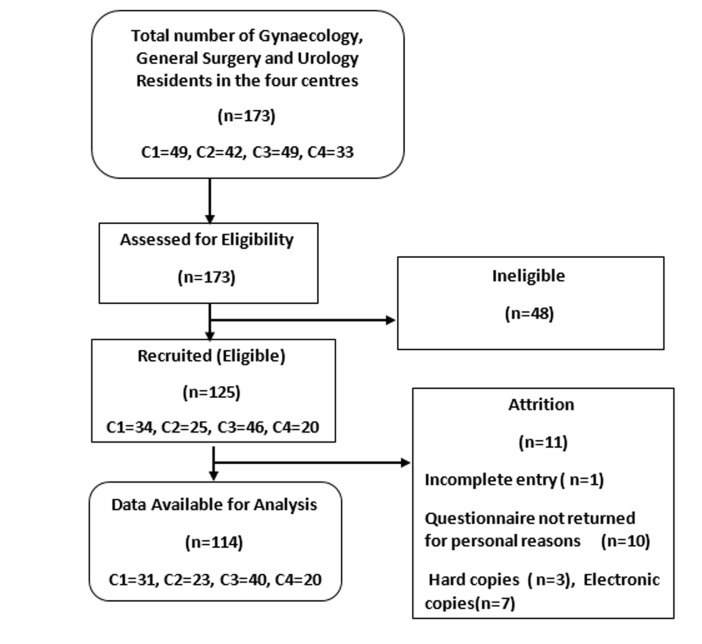
study flow diagram, Key-C1-National Hospital, Abuja C2- Federal Medical Centre, Abuja C3- University of Abuja Teaching Hospital, Abuja C4-Garki Hospital/Nisa Premier Hospital, Abuja

**General characteristics of the study population:** a total of 125 questionnaires were distributed of which 115 were retrieved, giving a response rate of 92%. One questionnaire had incomplete entries and was excluded from the final analysis (Figure 1). Of the respondents included in the final analysis, 31 (27.2%) were from National Hospital, 23 (20.2%) were from Federal Medical Centre, 40 (35.1%) were from the University of Abuja Teaching Hospital and 20(17.5%) from Garki Hospital Abuja. Gynaecology residents accounted for 84 (73.7%) while 20 (17.5%) and 10 (8.8%) of the respondents were from general surgery and urology respectively. The mean age of the respondents was 36.17±4.62 years while the mean duration of training was 53.91±28.02 months. There were 47 (56%), 15 (75%) and 9 (90%) males in gynaecology, general surgery and urology respectively. Fifty-five (48.2%) of the respondents were registrars. Of the 114 respondents, 110 (96.5%) reported that endoscopy is done in their centres, while four (3.5%), two residents each from National Hospital and University of Abuja Teaching Hospital reported otherwise.

The average number of endoscopic surgeries in the units per month was reported by 28 (34.6%) of gynaecology trainees as less than five, five to ten by 30 (37%) and more than ten by 23 (28.4%). In general surgery, less than five cases were reported by ten (52.6%), five to ten by six (31.6%) and more than ten by three (15.8%), two (20%) of the urology trainees reported less than five cases while five (50%) and three (30%) reported five to ten and more than ten cases per month respectively. In terms of competence 32 (28.1%) residents had only observed a procedure, 61 (53.5%) had assisted with surgeries, 16 (14%) had achieved competence for diagnostic procedures only and five (4.4%) had performed both diagnostic and operative procedures. Overall, 18 (15.8%) were satisfied with the state of endoscopy practice at their center while 96 (84.2%) were not satisfied. In terms of satisfaction with the level of endoscopy training received, four (3.5%) were satisfied, 17 (14.9%) had a neutral position, 55 (48.2%) were unsatisfied and 38 (33.3%) were very unsatisfied. None was very satisfied. All the residents considered training in endoscopy as a relevant component of the residency training program. Only 12 (10.5%) reported that they have had some formal training in endoscopy outside their work place, while 102 (89.5%) have had no formal training. Comparison of competence between those that had received training and those yet to receive training gave a p-value of 0.05 (Fisher 6.69).

Of those that have had training, 10 (83.3%) residents had basic training in Nigeria while two (16.7%) had advanced training within Nigeria but none has had any form of training outside Nigeria. Eighty-one (79.45%) of those yet to have a training were willing to self-sponsor their training while 72 (70.6%) desired government or institutional sponsorship for training, and 52 (51.0%) were willing to have both options. One Hundred and nine (95.6%) looked forward to having post-fellowship training in endoscopy while 5 (4.4%) desired otherwise. None of the respondents reported the availability of an endoscopy skills lab in their centre. When compared between the two genders, there were no statistically significant difference in competence (Fisher 4.66, P=0.18), perception of the relevance of endoscopy (Fisher 7.13, P=0.51), satisfaction with endoscopy training (Fisher-0.46, P=0.97), formal training (Chi square-2.43, P=0.12) and desire for post-fellowship training (Fisher 1.11, P=0.36). Comparison of competence between registrars and senior registrars showed a statistically significant difference (Fisher 51.81, P < 0.001) but there was no statistically significant difference in the perception of the relevance of endoscopy (Fisher 4.36, P=0.17), satisfaction with endoscopy training (Fisher-5.95, P=0.11, formal training (Chi-square-2.90, P=0.88) and desire for post-fellowship training (Fisher 1.67, P=0.37). The rest of the results are presented as tables and figures.

**Demographic characteristics of the respondents by specialty:** the detailed baseline demographics of the respondents by speciality are shown in Table 1.

**Table 1:** baseline demographics of the respondents

Variable	Gynaecology n=84	General Surgery n=20	Urology n=10
**Mean Age ± SD (years)**	36.57±4.95	34.5±3.52	36.10±2.7
Mean Duration of Training ± SD (months)	51.66±27.32	55.60±32.39	69.30±21.02
**Centre**			
National Hospital	20(23.8%)	6(30.0%)	5(27.2%)
Federal Medical Centre	16(19.0%)	6(30.0%)	1(10.0%)
University of Abuja	28(33.3%)	8(40.0%)	4(35.1%)
Garki Hospital	20(23.8%)	0(0%)	0(0%)
**Cadre**			
Registrar	45(53.6%)	9(45.0%)	1(10.0%)
Senior Registrar	39(46.4%)	11(55.0%)	9(90.0%)
**Gender**			
Male	47(56.0%)	15(75.0%)	9(90.0%)
Female	37(44.0%)	5(25.0%)	1(10.0%)

SD- Standard Deviation

**Perception of the resident doctors of limiting factors to endoscopy practice and their expectations:** this is shown in Table 2. The most commonly perceived limiting factor was lack of funding (66.7%), most of the resident doctors (85.1%) desired the incorporation of endoscopy training into the residency training curriculum and 90.4% desired hands-on experience during a training program.

**Figure 2:**
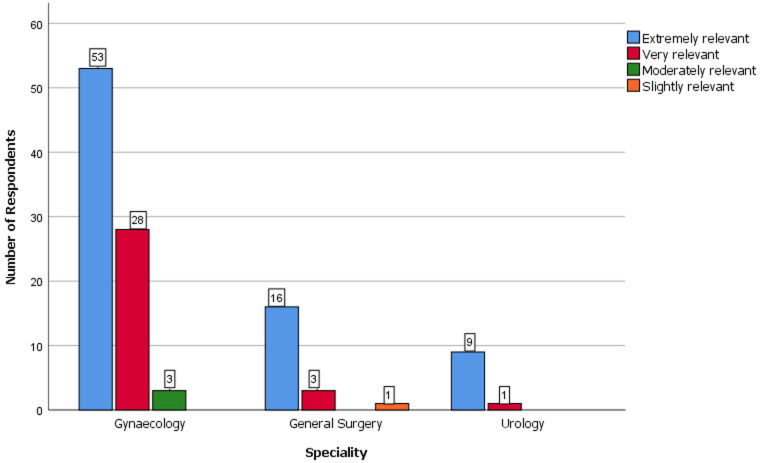
clustered bar chart showing the perception of the relevance of endoscopy

**Figure 3:**
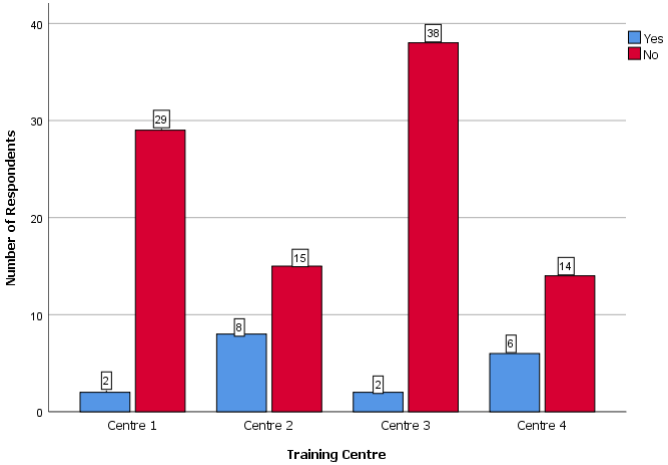
clustered bar chart showing the satisfaction of respondents with endoscopy practice in their training centre

**Figure 4:**
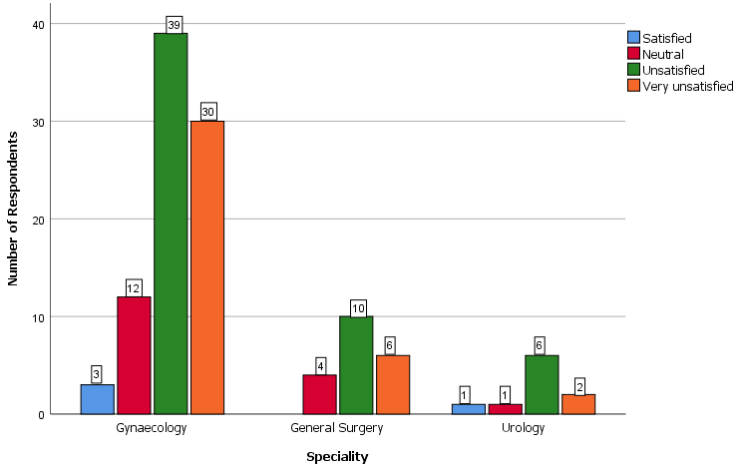
clustered bar chart showing the level of satisfaction of the respondents with endoscopy training in the different specialties

**Figure 5:**
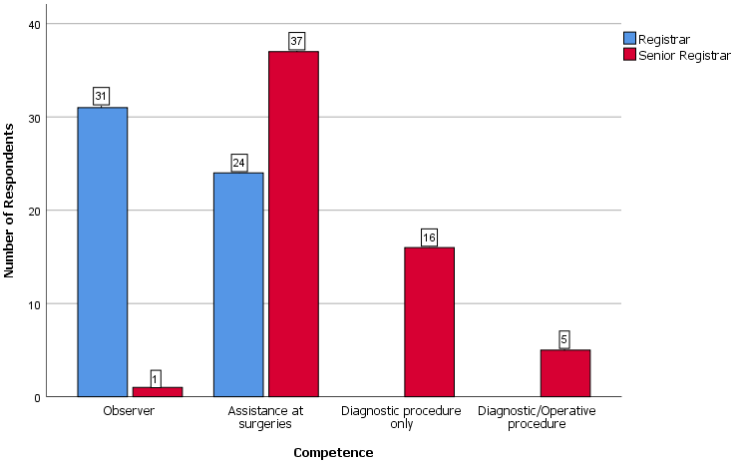
clustered bar chart showing the level of competence of the respondents according to cadre

**Comparison of the level of competence, perception of endoscopy training and level of satisfaction with endoscopy training of the resident doctors by specialty, training centre and cadre:** the comparison of key variables between the gynaecological and surgical specialties is shown in Table 3. There were no statistically significant differences in the level of competence, level of satisfaction, presence of formal training and desire for post-fellowship training (P>0.05). The difference in the perception of the relevance of endoscopy practice was however statistically significant (P=0.04). Figure 2 shows the perception of the resident doctors of the relevance of endoscopy across the three specialties. Most of the respondents considered endoscopy as extremely relevant. None considered it as not relevant. The level of satisfaction of respondents with endoscopy practice in their centers is shown in Figure 3. Most of the respondents expressed lack of satisfaction with the state of endoscopy practice. Figure 4 shows the level of satisfaction of the respondents with endoscopy training by speciality. Most of the respondents were unsatisfied with the state of training in their speciality. A few resident doctors in gynaecology and urology however expressed some level of satisfaction. The level of competence of the respondents according to cadre is shown in Figure 5. Only a few senior registrars had attained proficiency in operative endoscopy. Most of the registrars however had either only observed or assisted at surgeries.

**Table 2:** limiting factors to endoscopy practice/training and expectations of the respondents

Variable	Frequency (%)
**Limiting factors to endoscopy training and practice**	
Lack of specialist endoscopy surgeons/unit	50(43.9%)
Lack of endoscopy equipment	63(55.3%)
Lack of endoscopy skills lab	75(65.8%)
Lack of funding for training	76(66.7%)
Lack of dedicated theatre space	37(32.5%)
Cases not amenable to endoscopic surgery	13(11.48%)
**Expectations for endoscopy training and practice**	
Incorporation into the Residency training curriculum	97(85.1%)
Fully equipped endoscopy suite	74(64.9%)
Presence of endoscopy skills lab	83(72.8%)
Funding and leave to attend endoscopy training	72(63.2%)
Presence of endoscopy unit/specialist surgeon	68(59.6%)
**Expectations from a training program**	
Didactic lectures	47(41.2%)
Use of simulators	76(66.7%)
Use of animal models	54(47.4%)
Hands on (Live surgeries)	103(90.4%)
Objective assessment at end of training	81(71.1%)

**Table 3:** comparison of key variables between the gynaecological and surgical specialties

Variable	Gynaecology n=84	General Surgery/Urology n=30	Test Statistic	P value
**Competence**				
Observer	28(33.3%)	4(13.3%)	5.31^†^	0.17
Assistance at surgeries	41(48.8%	20(66.7%)		
Diagnostic procedure only	12(14.3%)	4(13.3%)		
Diagnostic/Operative	3(3.6%)	2(6.7%)		
**Relevance**				
Extremely relevant	53(63.1%)	25(83.3%)	8.35^†^	0.04*
Very relevant	28(33.3%)	4(13.3%)		
Moderately relevant	3(3.6%)	0(.0%)		
Slightly relevant	0(0%)	1(3.3%)		
Not relevant	0(0%)	0(0%)		
**Satisfaction**				
Very satisfied	0(0%)	0(0%)	0.85^†^	0.84
Satisfied	3(3.6%)	1(3.3%)		
Neutral	12(14.3%)	5(16.7%)		
Unsatisfied	39(46.4%)	16(53.3%)		
Very unsatisfied	30(78.9%)	8(21.7%)		
**Formal training**				
Yes	11(13.1%)	1(3.30%)	2.24‡	0.14
No	73(86.9%)	29(96.7%)		
**Desire for post fellowship training**				
Yes	80(95.2%)	29(96.7%)	0.11^†^	0.74
No	4(4.8%)	1(3.3%)		

† - Fisher’s Exact Test, ‡ - Pearson’s Chi Square, * - Statistically significant

## Discussion

This study sought to appraise the perception of and level of utilization of endoscopy by resident doctors in gynaecology, general surgery and urology in four training centres in Abuja. Our study showed more respondents in gynaecology, when compared to surgical residents. This is unlike the findings by De Win *et al*. in Belgium where there were more general surgeons [17]. The smaller number of responding surgical trainees (urology and general surgery) in our study can be explained by the subspecialisation of residents in surgery. This is unlike their gynaecology counterparts who are not as subspecialised during training thus availing for more respondents. There were more males in this study compared to females across all specialties when compared to a similar study in which there were more females in gynaecology [17]. This may be explained by the fact that surgical specialties are usually considered to be incompatible with the female gender in this part of the world quite unlike what obtains in the western world. The average age of the respondents is higher than that of respondents in a similar study in Belgium [17]. This may be because of difficulty in securing residency training positions in Nigeria. The average duration of residency of our respondents was 53.91±28.02 months. This is similar to reports by Majmudar *et al*. and Gabriel *et al*. in the UK and Germany where most of the residents had spent up to 36 months and 3.2 years in the residency training respectively [12,13]. All the respondents rated endoscopy training as relevant, this compares to reports from Germany by Gabriel *et al*. in which 90% and 80% of respondents considered laparoscopy and hysteroscopy training as important respectively [13]. Likewise in the study by Sklubeny *et al*. on general surgeons in Canada, 95.7% believed that endoscopy is an important skill for general surgeons to possess [16].

Most respondents (84.2%) expressed dissatisfaction with the level of endoscopy practice in their different centres. This is unlike the findings from a study in the UK that showed higher level of satisfaction [18]. Likewise in Germany, Gabriel *et al*. reported that only 23% of the gynaecology trainees were inadequately satisfied with the rest expressing some degree of satisfaction [13]. However, other studies in the UK and Canada showed that 69% of trainee gynaecologists and nearly half of the general surgeons (46.4%) were not satisfied with the level of expertise acquired [12,16]. The higher level of dissatisfaction in this environment could be linked to poor exposure to endoscopy practice. The findings from this study concur with findings by Balogun *et al*. on general surgery trainees that the average resident doctor only witnessed about one to four laparoscopic procedures throughout their entire rotations in general surgery [11]. Similar findings was reported by Ijah *et al*. in their study in Port Harcourt, Nigeria [10]. Only 10.5% of the participants reported that they have had formal training in endoscopy outside their workplace. This is similar to a study by Balogun *et al*. in which 90.7% were yet to have training in endoscopy [11]. Even in developed countries, there is still a relatively low attendance rate for structured endoscopy courses. Nasralla *et al*. reported that only 45% of the general surgery trainees in England had attended any course on endoscopy with about 10% citing difficulty in getting placement and exorbitant cost as hindrances [19]. Similarly, in Belgium, De win *et al*. reported that only 26% of gynaecology residents, 36% of general surgery residents, and 52% of urology residents received extra laparoscopy training not organized by their training program or university [17].

A good number (79.5%) of the resident doctors in this study were however willing to sponsor themselves for training if given the opportunity. This concurs with reports by Gabriel *et al*. in Germany [13]. Improvement of skills has been demonstrated as a by-product of training but our study shows a gross deficiency in training. The statistically significant difference in competence between the junior and senior residents also affirms the fact that training is very vital to improvement in endoscopy service delivery [5,17]. A great number of the respondents were yet to achieve competence in operative endoscopy. This is similar to findings from a similar study done in Nigeria [11]. Conversely reports by Majmudar *et al*. from their study in the UK showed that almost all had achieved competence in level 1 laparoscopic procedures, even though experience with advanced procedures was still limited [12]. Likewise, a survey of general surgery trainees in Belgium showed that all fifth- and sixth-year trainees had performed more than 25 laparoscopic appendectomies and more than 25 laparoscopic cholecystectomies without supervision [17].

All the respondents reported a lack of endoscopy skills lab in their centre. This is similar to the findings from a study on general surgery resident doctors [11]. This is unlike reports from Belgium where a substantial proportion of the residents stated they have access to a laparoscopic skills lab [17]. In a UK study, 34% of trainees reported that they had worked on a simulator in their training [12]. The limited availability of simulators in this environment may explain further why the exposure and utilization of endoscopy in this locality are low. Most residents (66.7%) cited lack of funding as the greatest obstacle to training and practice in their centre. This is similar to findings from a similar study in Nigeria [11]. However in a study in the UK, 87% of respondents believed that the most common factor hindering training was the inability to be the primary surgeon [12]. Studies done in the USA showed marked improvement of endoscopic training over time [20,21]. This is in sharp contrast to the state of practice in Nigeria as there has not been substantial improvement in the level of endoscopy practice in residency training centres over the years.

The most common expectation of the residents was the incorporation of structured endoscopy training into the residency training curriculum (85.1%) while 72.8% desired the presence of an endoscopy skills lab. In a study in the Netherlands, most of the residents(86.9%) opined that every surgical hospital department should have a surgeon specialized in laparoscopic surgery [15]. The presence of specialist endoscopy surgeon and a skills lab was considered as important for teaching endoscopy skills by respondents in a study in the UK [12]. A study by Shore *et al*. shows that a comprehensive simulation-based training curriculum leads to improvement in knowledge and operating room technical performance when compared with conventional residency training [22].

Almost all of the respondents desired to pursue further training in endoscopy at the post-fellowship level. Balogun *et al*. reported that 44 (81.0%) of respondents in their study desired a future practice in laparoscopy [11]. This willingness to pursue further training in endoscopy despite the prevailing challenges shows a high level of motivation. A sound residency endoscopy training foundation will go a long way in enhancing further training experience. Endoscopy is majorly private-sector driven in Nigeria, yet the bulk of residency training occurs in the public health sector. Unfortunately, not enough has been done in providing endoscopy facilities in government-owned tertiary and secondary health care centres in order to boost endoscopy training. There are so many derivable benefits in terms of improvement in endoscopy skills if training is optimised in residency training institutions [4]. The provision of an enabling environment will improve training exposure and experience of endoscopy during the residency training program.

**Limitations:** this study is a questionnaire-based cross-sectional study and as such is prone to responder and recall bias, the results therefore, may not be completely representative of the true state of events.

## Conclusion

This study showed a poor level of exposure to endoscopy training and high level of dissatisfaction with endoscopy practice among resident doctors in the three specialties across the four residency training centres in Abuja. A very high expectation of the residents for improved services was also observed. It is recommended that structured endoscopy training should be incorporated into the residency training curriculum. Training centres should also be well equipped with an endoscopy operative suite and endoscopy skills lab to aid learning and this should be a prerequisite for accreditation of training centres. Replication of this study in other localities within the country is essential.

### What is known about this topic

- Endoscopy is an attractive option for the surgical management of gynaecological and surgical diseases;
- Competence in endoscopy is developed through structured training;
- Training is a crucial to the development of a robust endoscopy practice.

### What this study adds

- The level of exposure of gynaecology and surgery residency trainees in Abuja to endoscopic surgery practice and training;
- The perceived limitations to endoscopy practice and training in residency training centres in Abuja;
- The specific expectations of gynaecology and surgery residency trainees in Abuja for improved endoscopy training.
